# Regional limb cooling for the prevention of chemotherapy-induced toxicities: a narrative review

**DOI:** 10.1007/s00520-025-09689-y

**Published:** 2025-07-10

**Authors:** Teja Mallela, Lily Kaufman, Brittany Dulmage

**Affiliations:** 1 The Ohio State University College of Medicine, 540 Officenter Place, Suite 240, Gahanna, Columbus, OH 43230 USA; 2 Department of Dermatology, The Ohio State University Wexner Medical Center, Columbus, OH USA

**Keywords:** Regional limb cooling, Cryotherapy, Chemotherapy-induced peripheral neuropathy, Hand-foot syndrome, Nail changes, Oncodermatology

## Abstract

**Purpose:**

Chemotherapy-induced peripheral neuropathy (CIPN), hand-foot syndrome (HFS), and nail changes are common adverse effects associated with chemotherapy, significantly impacting the quality of life of cancer patients, and effective interventions remain an unmet need in oncologic care. Regional limb cooling is an emerging non-pharmacological approach used to prevent or reduce the severity of these adverse side effects. Although the awareness of regional limb cooling is rather prevalent, the overall use and efficacy of this non-pharmacological therapy in preventing chemotoxicities is not universally agreed upon. This paper reviews the current evidence regarding the use, efficacy, limitations, and safety of regional limb cooling in preventing or reducing CIPN, HFS, and nail changes in clinical settings.

**Methods:**

A literature review was conducted using the PubMed database to identify the most recent English articles published between 2004 and 2024 regarding the use of regional limb cooling to prevent CIPN, HFS, and nail changes.

**Results:**

Based on the reviewed studies, regional limb cooling can potentially be a low-risk, non-pharmacological intervention that can prevent and reduce common and debilitating chemotherapy-induced toxicities. Although the studies described in this paper demonstrate encouraging results, there is still notable variability in efficacy.

**Conclusion:**

This underscores the need for randomized controlled trials, device innovation, and standardized protocols in regional limb cooling to properly demonstrate its potential for alleviating these common, debilitating conditions and enabling patients to adhere to their chemotherapy regimens more comfortably. This would thereby enhance overall cancer treatment tolerability and lead to safer, more patient-centered chemotherapy management.

## Introduction

Chemotherapy-induced peripheral neuropathy (CIPN), hand-foot syndrome (HFS), and nail changes are common adverse effects associated with chemotherapy, significantly impacting the quality of life of cancer patients. Effective preventative interventions for adverse effects of treatment remain an unmet need in oncologic care. Regional limb cooling, or cryotherapy, is an emerging non-pharmacological approach used to prevent or reduce the severity of the adverse side effects caused by chemotherapy. Limb cooling is thought to work by selectively decreasing the blood flow in peripheral tissues and, hence, reducing the drug uptake in these areas. Although the awareness of regional limb cooling is rather prevalent, the overall use and efficacy of this non-pharmacological therapy in preventing chemotoxicities is not universally agreed upon. This paper reviews the current evidence regarding the use, efficacy, limitations, and safety of regional limb cooling in preventing or reducing CIPN, HFS, and nail changes in clinical settings.

## Methods

A literature review was conducted for the most recent articles regarding the use of regional limb cooling to prevent CIPN, HFS, and nail changes caused by chemotherapy. The authors searched the PubMed database for English articles that have been published between 2004 and 2024. This was not a systematic review. Our narrative review aims to characterize the existing literature and summarize the use of regional limb cooling for chemotherapy-induced toxicities. The reviewed articles often differed in methodology and may not be directly comparable. Additionally, this review is solely based on previously conducted studies and contains no new studies or analyses.

### Summary of regional limb cooling methods

Various regional limb cooling methods have been used for the prevention of chemotherapy-induced toxicities. Within the literature, the most common and rudimentary method usually is some variation of cooled gloves and socks. When using cooled gloves or socks, these garments are often frozen in temperatures between − 20 and − 30 °C and applied to the hands and feet 15–30 min before chemotherapy treatment begins and continues for an additional 15–30 min after the treatment has concluded [21, 52]. Additionally, some studies noted changing the frozen gloves and socks every 15 min to maintain optimal cooling [9]. This may be burdensome to patients who need to bring cooling supplies with them to infusions and may require assistance from a family member or nursing staff to help change garments. To mitigate the need for changing gloves and socks, continuous cooling therapy devices maintain a constant temperature over longer periods; these devices have been shown to achieve greater temperature stability when compared to traditional frozen gloves and socks [11]. Barriers to the uptake of this form of cooling are the need for infusion centers to have equipment available and the time to pre-cool and post-cool, which may increase the overall time patients utilize an infusion chair. Overall, regional limb cooling is well tolerated, with only minor side effects noted, such as mild pain, burning, or stinging [3, 16]. Severe adverse effects from regional limb cooling are extremely rare, but it is important to note that two cases of frostbite were reported with its use [14]. Interestingly, a study found that adding compression to limb cooling might improve the preventative effects [4]. This method, known as cryocompression, combines regional limb cooling with compression of the hands and feet. One study utilized smaller, tighter gloves as a compression method while administering continuous cooling therapy [6]. Other studies have used pneumatic compression that cycled between 5 and 15 mmHg of pressure through wraps on all four limbs [4]. It is unclear whether cryocompression is superior to cooling alone, but cryocompression and all the other cooling variations reflect the ongoing efforts to prevent chemotherapy-induced toxicities.

### Chemotherapy-induced peripheral neuropathy

Chemotherapy-induced peripheral neuropathy (CIPN) is a prevalent, and often irreversible [27], side effect of cancer treatments, particularly taxane-based chemotherapies like paclitaxel [24]. Various strategies, including regional limb cooling, have been explored to mitigate CIPN by inducing vasoconstriction, which may reduce chemotherapy exposure in peripheral nerves. This section reviews the effectiveness, safety, and tolerability of regional limb cooling in preventing CIPN and provides evidence suggesting that it could be used as a non-pharmacologic preventive intervention in treating cancer patients.

According to several studies, regional limb cooling can lower the incidence and severity of CIPN [35]. One study reported significantly lower incidences of grade 2 or higher peripheral neuropathy in paclitaxel-treated patients using cryotherapy (*p* < 0.05) [42]. Another study found that cryotherapy reduced both objective and subjective neuropathic symptoms, significantly improving tactile sensitivity and motor function [17]. Sundar et al. [45] observed continuous flow hypothermia correlated with preserving motor nerve amplitudes over 6 months. Additional studies support these findings. Kanbayashi et al. [23] found comparable efficacy between cryotherapy and compression therapy for preventing neuropathy, while noting that mean fingertip temperatures were significantly lower in the cryotherapy group (22.3 °C) compared to the compression group (27.9 °C; *p* < 0.0001) after treatment. However, Griffiths et al. reported no significant differences in neuropathy between cryotherapy-treated and untreated groups. However, in Griffiths et al.’s study, it is important to note that 34% were unable to tolerate cryotherapy, and 21% declined further participation. Thus, only 24% of participants completed the final assessments, which may have contributed to the lack of observed efficacy [16]. Ruddy et al. [41] concluded that cryotherapy had no significant effect on sensory scores over 12 weeks of paclitaxel, though it was well tolerated. Another study by Rosenbaek et al. [40] observed that prophylactic cryotherapy reduced CIPN-related dose limitations, allowing more patients to complete the planned paclitaxel regimen. In systematic reviews [8], cryotherapy’s efficacy remains variable. Bailey et al. [2] reported mixed results across studies, noting that using frozen gloves and socks often reduced CIPN symptoms but with some inconsistencies due to heterogeneous study designs and cooling methods. Peyton and Fischer-Cartlidge [37] and Accordino et al. [1] recommended more extensive studies to better assess cryotherapy’s effectiveness. Cryocompression has shown potential for enhancing cryotherapy effects; Bandla et al. [4] demonstrated better cooling and adherence with cryocompression compared to traditional methods.

The safety profile of cryotherapy in CIPN prevention is well-documented [53, 54]. Bandla et al. [3] showed that continuous flow cooling was well tolerated in healthy subjects, with only minor side effects like transient erythema and numbness. Similarly, Bandla et al. [4] reported that cryocompression allowed for sustained cooling without significant adverse effects. Griffiths et al. [16] observed mild discomfort (i.e., mild pain, burning, or stinging) as the primary side effect, leading to a 34% treatment discontinuation rate. To address comfort issues, Binder et al. [5] developed a cryo-compression device that allowed for more comfortable, sustained cooling, potentially improving adherence. Sphar et al. [44] recommended using controlled cryotherapy systems to address variability and improve safety outcomes. However, studies like Oneda et al. [36] reported that while regional limb cooling was generally safe, mild side effects such as pain, burning, or stinging could decrease adherence. When reviewing the meta-analyses, regional limb cooling showed a low risk of severe adverse events, although discomfort during use remains a barrier to widespread use. For example, Griffiths et al. [16] and Ruddy et al. [41] reported high tolerance in controlled conditions, confirming the safety profile of limb cooling despite discomfort issues. According to a 2021 study by Bailey et al. [2], significant adverse events were uncommon, but discomfort-related discontinuation was widespread, highlighting the need for additional device innovation to enhance patient experience. Although regional limb cooling is generally very well tolerated [47], frostbite has been reported in two cases during off-label use [14]. Therefore, it is crucial that careful monitoring occurs during regional limb cooling treatment.

Because of the need for more conclusive evidence [8, 26], the American Society of Clinical Oncology (ASCO) does not formally recommend regional limb cooling as a preventative measure against CIPN. However, based on these studies, regional limb cooling has shown the potential to be safe and could be used to lower the incidence and severity of CIPN. Additionally, Rosenbaek et al. [40] found that cryotherapy allowed patients to complete their chemotherapy regimens by reducing CIPN symptoms, supporting the intervention’s role in enhancing treatment tolerability. Still, there is a need for more conclusive evidence [32], and larger [43, 46], long-term randomized controlled trials are being conducted to validate the efficacy and best practices for regional limb cooling. As cooling technology continues to develop, its use may be considered in oncology as a low-risk option to manage chemotherapy toxicities like CIPN.

### Hand foot syndrome

**Fig. 1:**
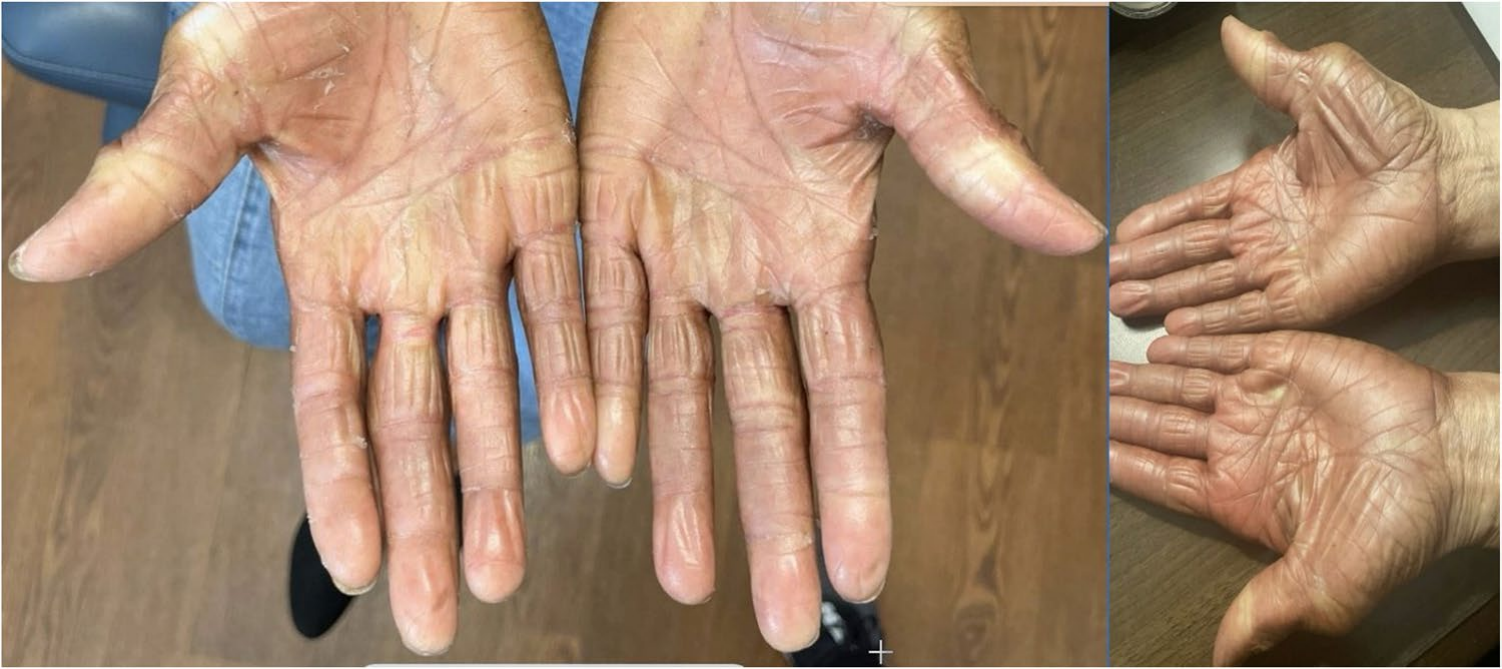
Hand-foot syndrome of the bilateral palms in two separate cancer patients on capecitabine

Hand-foot syndrome (HFS), also referred to as palmar-plantar erythrodysesthesia, is a common cutaneous side effect associated with chemotherapy, particularly with drugs like pegylated liposomal doxorubicin (PLD) [56]. HFS presents as redness, swelling, and pain on the palms of the hands and soles of the feet and, in severe cases, can lead to blistering and peeling of the skin (Fig. 1). Regional limb cooling has been explored as a potential preventative measure to mitigate the discomfort and interruptions in chemotherapy that HFS can cause. This section reviews the efficacy, safety, and tolerability of regional limb cooling based on relevant literature.

Studies demonstrate a range of efficacy outcomes for regional limb cooling in preventing HFS, with results often contingent on study design, patient populations, and concurrent interventions. Zheng et al. [55] showed a significant reduction in HFS incidence, with a mere 2% in patients receiving cooling compared to 40% in the control group. Similarly, Mangili et al. [29] noted that adding regional cooling to a premedication protocol with pyridoxine and dexamethasone significantly lowered HFS occurrence from 36 to 7.1%. Other studies support these findings but suggest a nuanced understanding of efficacy. Bun et al. [7], using frozen gloves and socks (FGS) during PLD administration, observed reduced severity and frequency of HFS among patients receiving regional cooling. However, the optimal cooling duration remains unknown, as PLD’s extended half-life may require prolonged cooling beyond chemotherapy sessions for maximum efficacy. Interestingly, some studies, such as Tanyi et al. [48] and Julius et al. [22] reported higher or equivalent rates of HFS in patients who used cooling therapy, challenging the efficacy of this intervention. The variability in efficacy in these studies could be due to differences in cooling methods, chemotherapy regimens, or patient populations.

The safety and tolerability of regional limb cooling vary among patients at risk for HFS, with most studies indicating favorable outcomes. Molpus et al. [33] reported high patient compliance and low toxicity when using wrist and ankle cooling with ice packs, with only 6% of patients experiencing severe HFS (grades 3–4). However, Bun et al. [7] observed that some patients discontinued FGS due to pain from cold exposure, which highlights potential discomfort in using extreme cooling methods. Additionally, Webster-Gandy et al. [50] noted that challenges exist, particularly the impracticability of extended cooling time, which most affects patients undergoing infusions with long blood circulation times or taking oral chemotherapy such as capecitabine, which carries a high risk of HFS.

Regional limb cooling offers a promising non-pharmacological approach to managing HFS in patients undergoing PLD therapy. Although there is conflicting evidence on its efficacy, many studies continue to support its role in reducing the incidence and severity of HFS with minimal tolerability issues. Future studies are needed to optimize cooling duration and comfort and explore adjunctive therapies such as steroids to enhance limb cooling efficacy. Large-scale randomized controlled trials are particularly needed to standardize protocols and clarify the role of regional limb cooling in HFS prevention.

### Nail changes

**Fig. 2:**
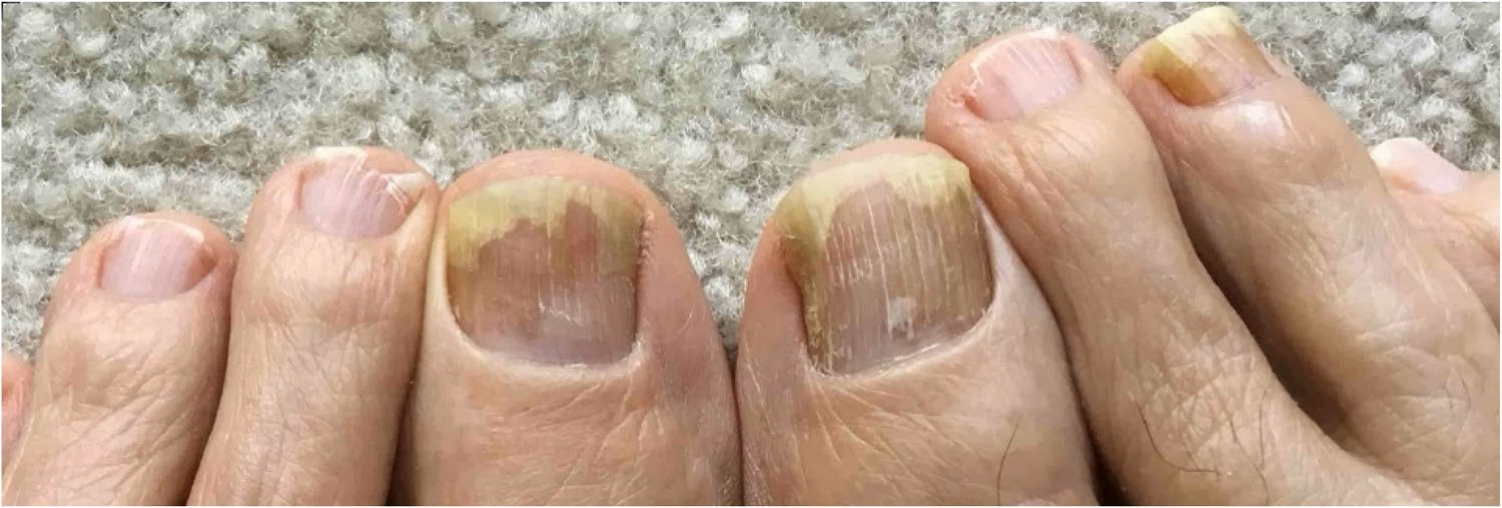
Bilateral onycholysis of the toe nails in a cancer patient on docetaxel

**Fig. 3:**
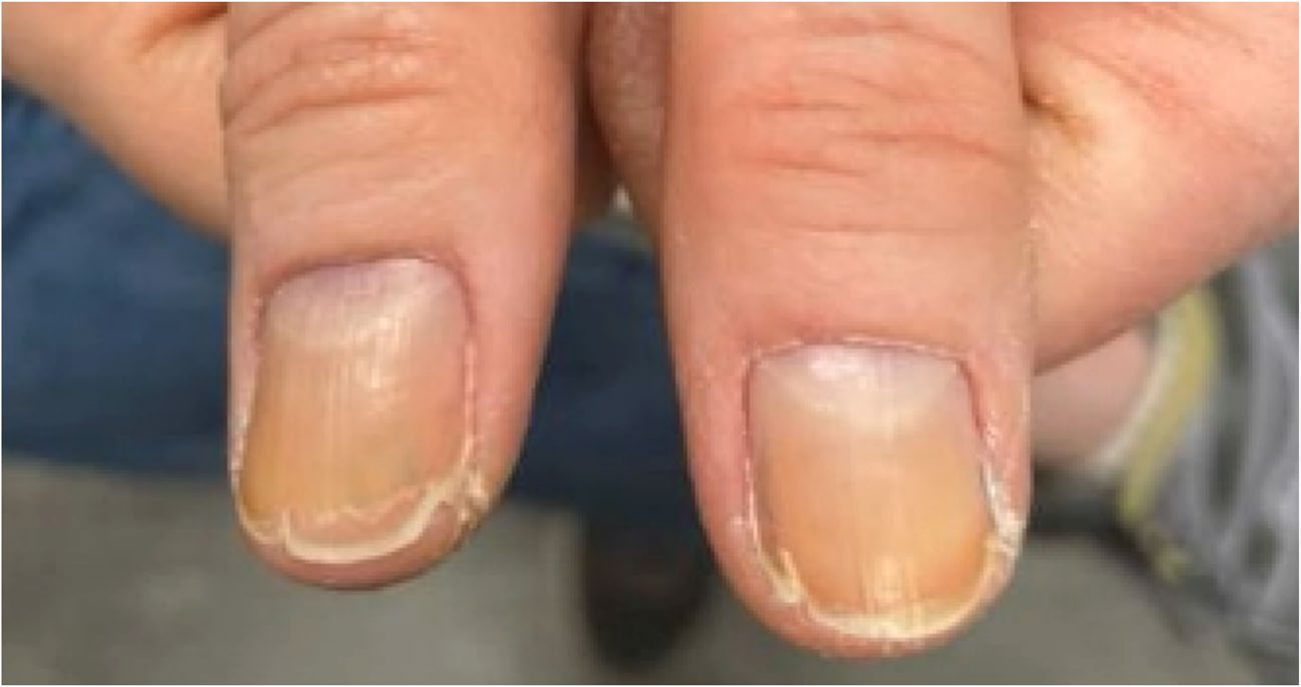
Bilateral onycholysis of the thumb nails in a cancer patient on pertuzumab, trastuzumab, and docetaxel

Nail toxicities, including onycholysis (Figs. 2 and 3), discoloration, and nail bed hemorrhages, are notable side effects of chemotherapy, particularly taxane-based regimens. These nail changes can impact the quality of life in cancer patients and affect their chemotherapy dosage and scheduling. Studies on regional limb cooling to prevent and reduce chemotherapy-induced nail changes have shown promise. This section reviews recent studies evaluating the efficacy, safety, and tolerability of cooling techniques to prevent nail toxicity.

Studies generally support the efficacy of regional limb cooling in reducing chemotherapy-induced nail toxicity. Johnson et al. [20] used the Hilotherapy machine to cool patients’ dominant hand and foot to approximately 10–12 °C. They observed reduced nail toxicity in the cooled extremities, although nail changes were not entirely prevented. Nonetheless, the extremities that were cooled exhibited less severe symptoms, supporting the efficacy of regional cooling [20]. Mazzega-Fabbro et al. [31] employed a more straightforward method, applying disposable ice packs to the hands and feet. This intervention significantly reduced grades 1 and 2 nail toxicity, demonstrating that even basic cryotherapy can protect against nail changes [31]. Likewise, Peyton and Fischer-Cartlidge [37] reported on studies utilizing frozen gloves and socks, which consistently reduced nail toxicity compared to uncooled extremities. In a separate approach, Morrison et al. [34] studied massage-based interventions and found reduced nail issues compared to cryotherapy. Morrison et al. proposed that the massaging stimulated certain vascular elements of the nail matrix, which helped mitigate nail changes; however, the exact mechanism is still in question. Huang et al. [18] conducted a meta-analysis of cryotherapy studies, highlighting a statistically significant reduction in nail toxicity among those receiving cryotherapy, reinforcing the efficacy of these interventions. Gilbar et al. reference a phase II matched case–control study and found a significant decrease in onycholysis in patient hands that underwent frozen glove therapy, with grade 2 onycholysis occurring in 0% of frozen glove-protected hands compared to 21% of control hands. Additionally, 89% of cooled hands had no onycholysis versus 49% in the control group [15]. This study, and subsequent studies, further supports frozen gloves as an effective preventive measure against taxane-induced nail changes [12, 13, 15].

Regional limb cooling is generally well tolerated, although cold intolerance is a notable concern affecting compliance. Johnson et al. [20] reported low discontinuation rates due to discomfort, indicating that the Hilotherapy method was well tolerated. Mazzega-Fabbro et al. [31] also noted that patients using disposable ice packs reported only mild to moderate pain, with no cases of severe discomfort or adverse effects. Peyton and Fischer-Cartlidge [37] reviewed studies that reported cold intolerance as a common barrier, with attrition rates ranging from 2 to 71%. Despite these variations, frozen gloves and socks were generally well received and associated with few severe adverse effects. Marks et al. and Jia et al. also highlighted the need for standardized protocols to improve patient comfort and provide consistent safety assessments across studies [19, 30]. Thomas et al. [49] explored the practicality of combining cryotherapy with a polyphenolic-rich nail balm in the UK, where commercially available gloves are less commonly used due to concerns about limited hand access for patients. They found that the nail balm alone significantly improved nail quality, with patients in the nail balm group experiencing a mean deterioration in Dermatology Life Quality Questionnaire (DLQQ) score of only 0.034 compared to 6.10 in the placebo group (*p* < 0.001) [49]. This suggests that polyphenolic-rich nail balm could act as an additional or alternative supportive intervention when cryotherapy is impractical.

Overall, regional limb cooling shows promise as a preventive strategy for chemotherapy-induced nail toxicity. Various methods are effective in reducing the incidence and severity of nail changes. Despite cold intolerance affecting patient compliance, several methods of limb cooling have shown to be safe and well tolerated. Although cold intolerance can affect compliance, these interventions are safe and well tolerated. Continued investigation into standardized protocols will benefit this approach and enhance patient quality of life during chemotherapy.

### Treatment barriers and future directions

Certain treatment barriers need to be considered when deciding to use regional limb cooling to prevent chemotherapy-induced toxicities. The most important barrier is the potential for patient discomfort that may come with prolonged cold exposure. For example, one study reported a 34% dropout rate due to the cold discomfort [16]. Additionally, patients with pre-existing conditions that could predispose them to cold discomfort, such as Raynaud disease, would contraindicate regional limb cooling [28]. This discomfort could result from non-uniform cooling, abrupt temperature gradients, or the lack of cooling adjustment based on tolerance at the individual level. There is also a logistical barrier due to the additional time it takes to implement cooling methods for patients in the fast-paced environment of a chemotherapy infusion clinic [51]. The logistical burden only intensifies with methods that require consistently changing frozen gloves and socks throughout the chemotherapy infusion [21]. The articles reviewed in this study also present heterogeneity in study designs and cooling methods utilized, which reflects the lack of standardized guidelines and varying rates of efficacy. This variability among studies makes it difficult to draw meaningful conclusions on the benefits of regional limb cooling—reducing the likelihood of universal application of this preventative therapy [38, 39].

Future directions include large-scale, randomized controlled trials that use a standardized cooling method in order to provide robust data on the efficacy and tolerability of regional limb cooling, similar to that which is available for scalp cooling. More conclusive data on its efficacy can lead to formal guidelines created by organizations, such as the ASCO, to promote the best practices for regional limb cooling [25]. Additionally, the development of more advanced cooling devices that are patient-friendly and efficient can help reduce patient discomfort and logistical barriers. Cryocompression wraps might offer a solution to these barriers. Bandla et al. and Binder et al. highlighted the potential for cryocompression wraps to reduce discomfort issues and achieve better cooling outcomes. Continuous cooling systems are a promising innovation as well [4, 5]. These innovations, alongside increased clinician awareness and education, could pave the way for the broader adoption of regional limb cooling as a standard preventive measure for CIPN, HFS, and nail changes.

## Conclusion

In conclusion, regional limb cooling has the potential to be a low-risk, non-pharmacological intervention that can prevent and reduce common and debilitating chemotherapy-induced toxicities such as CIPN, HFS, and nail changes (Table 1). By alleviating these side effects with limb cooling, patients can adhere to their chemotherapy regimens more comfortably and enhance overall cancer treatment tolerability. Although the studies described in this paper demonstrate encouraging results, there is still notable variability in efficacy, with some studies also noting the challenges in adherence due to discomfort induced by cold exposure. This emphasizes the need for randomized controlled trials, device innovation, and standardized protocols in regional limb cooling to improve patient experience and optimize cancer therapy outcomes. As we continue to understand the mechanisms of regional limb cooling and its optimal usage, this non-pharmacological intervention could play a meaningful role in supportive cancer care and ultimately lead to safer, more patient-centered chemotherapy management.

**Table 1:** Table summary of chemotherapy-induced toxicities

Toxicity	Clinical findings	Commonly associated chemotherapies	Summary of evidence	Adjunctive or alternative therapies
CIPN	Tingling, numbness, burning, and motor weakness in extremities, potentially impacting daily activities and quality of life. Symptoms are often dose-dependent and can persist long-term	Taxanes (e.g., paclitaxel, docetaxel), platinum-based agents (e.g., oxaliplatin), and alkaloids	Evidence supports the efficacy of regional limb cooling in reducing CIPN severity and incidence. Studies, including those by Sato et al. and Hanai et al., found significant reductions in neuropathy with cryotherapy. However, evidence is inconsistent, with some studies, like Griffiths et al., reporting no significant effect. Safety is well-documented, though discomfort remains a barrier to adherence	Physical therapy, acupuncture [10], and pharmacologic agents such as duloxetine. Additionally, cryocompression showed better cooling adherence and potential efficacy [4]
HFS	Redness, swelling, and pain on palms and soles; severe cases include blistering and peeling, which may lead to treatment delays or dose reductions	Pegylated liposomal doxorubicin (PLD), capecitabine, fluorouracil	Regional limb cooling has shown promise in reducing HFS severity. Studies by Zheng et al. and Mangili et al. demonstrated significant reductions in HFS incidence. However, variability in cooling protocols and patient populations affects overall outcomes	Evidence suggests potential adjunctive benefits with premedication protocols, such as pyridoxine, topical steroids, urea-based creams, and dexamethasone
Nail changes	Onycholysis, discoloration, subungual hemorrhages, and nail bed pain, often resulting in functional and aesthetic concerns for patients	Taxanes (e.g., paclitaxel, docetaxel)	Studies conducted by Johnson et al. and Mazzega-Fabbro et al., support the efficacy of regional limb cooling in reducing nail toxicities. Frozen gloves and socks, as well as continuous flow cooling devices, reduced the incidence and severity of nail changes. Variability in methods and lack of standardized protocols remain challenges in determining efficacy	Nail balms rich in polyphenols, protective nail lacquers, and patient education on nail care

## Data Availability

No datasets were generated or analysed during the current study.
